# 1-(3-*Tert*-Butylphenyl)-2,2,2-Trifluoroethanone as a Potent Transition-State Analogue Slow-Binding Inhibitor of Human Acetylcholinesterase: Kinetic, MD and QM/MM Studies

**DOI:** 10.3390/biom10121608

**Published:** 2020-11-27

**Authors:** Irina V. Zueva, Sofya V. Lushchekina, Ian R. Pottie, Sultan Darvesh, Patrick Masson

**Affiliations:** 1 Arbuzov Institute of Organic and Physical Chemistry, Federal Research Center “Kazan Scientific Center of the Russian Academy of Sciences”, Arbuzov str., 8, 420088 Kazan, Russia; zueva.irina.vladimirovna@gmail.com; 2 Emanuel Institute of Biochemical Physics, Russian Academy of Sciences, Kosygin str. 4, 119334 Moscow, Russia; sofya.lushchekina@gmail.com; 3 Department of Chemistry and Physics, Mount Saint Vincent University, Halifax, NS B3M 2J6, Canada; ian.pottie@msvu.ca (I.R.P.); sultan.darvesh@dal.ca (S.D.); 4 Department of Chemistry, Saint Mary’s University, Halifax, NS B3M 2J6, Canada; 5 Department of Medicine (Neurology and Geriatric Medicine) & Medical Neuroscience, Dalhousie University, Halifax, NS B3H 4R2, Canada; 6 Neuropharmacology Laboratory, Kazan Federal University, Kremlevskaya str. 18, 480002 Kazan, Russia

**Keywords:** acetylcholinesterase, slow-binding inhibition, transition state analog, organophosphorus

## Abstract

Kinetic studies and molecular modeling of human acetylcholinesterase (AChE) inhibition by a fluorinated acetophenone derivative, 1-(3-tert-butylphenyl)-2,2,2-trifluoroethanone (TFK), were performed. Fast reversible inhibition of AChE by TFK is of competitive type with *K_i_* = 5.15 nM. However, steady state of inhibition is reached slowly. Kinetic analysis showed that TFK is a slow-binding inhibitor (SBI) of type B with K_i_* = 0.53 nM. Reversible binding of TFK provides a long residence time, *τ* = 20 min, on AChE. After binding, TFK acylates the active serine, forming an hemiketal. Then, disruption of hemiketal (deacylation) is slow. AChE recovers full activity in approximately 40 min. Molecular docking and MD simulations depicted the different steps. It was shown that TFK binds first to the peripheral anionic site. Then, subsequent slow induced-fit step enlarged the gorge, allowing tight adjustment into the catalytic active site. Modeling of interactions between TFK and AChE active site by QM/MM showed that the “isomerization” step of enzyme-inhibitor complex leads to a complex similar to substrate tetrahedral intermediate, a so-called “transition state analog”, followed by a labile covalent intermediate. SBIs of AChE show prolonged pharmacological efficacy. Thus, this fluoroalkylketone intended for neuroimaging, could be of interest in palliative therapy of Alzheimer’s disease and protection of central AChE against organophosphorus compounds.

## 1. Introduction

Fluoroalkylketones (FAK) are potent inhibitors of acetyl cholinesterase (AChE, ES.3.1.1.7) and butyrylcholinesterase (BChE, EC.3.1.1.8) [1,2,3,4]. A characteristic of inhibition by these compounds is the slow establishment of equilibrium between enzyme and inhibitor. This process is called slow-binding inhibition (SBI). Unlike classical reversible inhibitors for which equilibrium establishes within microseconds, in SBI, establishment of equilibrium may take seconds, minutes or hours. Three types of SBI have been described: (1) type A is characterized by a single step mechanism with slow *k_on_* and *k_off_*; (2) type B is a two-step mechanism: after rapid formation of a first enzyme-inhibitor complex, a slow induced-fit step occurs; (3) type C results from the existence of several enzyme forms in slow equilibrium that determine a slow conformational selection for inhibition [5].

**Figure 1:**
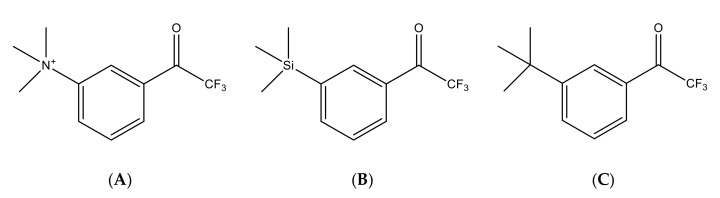
Chemical structure of related trifluoromethylketone molecules: (**A**): 1-[*3*-(trimethylamino)phenyl]-2,2,2-trifluoro-1-ethanone (TMTFA); (**B**): 2,2,2-trifluoro-1-[*m*-(trimethylsilyl)phenyl]-1-ethanone (Zifrosilone); (**C**): 1-(3-*tert*-butylphenyl)-2,2,2-trifluoroethanone (TFK).

Kinetic analysis of AChE inhibition by 1-[*3*-(trimethylamino)phenyl]-2,2,2-trifluoro-1-ethanone (TMTFA) (Figure 1A) showed that this compound is a slow-binding inhibitor of type A for *Torpedo californica* AChE (*K_i_* = 15 × 10^−15^ M; *t*^diss^_1/2_ = 2.8 h) [3] whereas it a slow-binding inhibitor of type B for electric eel (*Electrophorus electricus*) AChE (*K_i_* = 1.3 × 10^−15^ M; *t*^diss^_1/2_ = 19 h) [3,4]. Later, the X-ray structure of *Torpedo californica* AChE-TMTFA complex (PDB ID 1AMN [6]) showed that the tight interactions between the enzyme and inhibitor are similar to interactions that take place in the enzyme active center with acetylcholine in the transition state [6]. Thus, FAK, first considered as quasi-substrate inhibitors, are in fact transition state analogues.

In early 1990s, when AChE inhibitors started to be developed for the palliative treatment of Alzheimer disease (AD), a related silyl compound, Zifrosilone (Figure 1B) [7] was considered as a promising anti-AD symptomatic drug. Though pharmacokinetic/pharmacodynamic (PK/PD) studies were advanced, human clinical trials were discontinued in mid-90s [8,9,10]. Yet, incorporation of a silicone atom does not induce additional toxicity to molecules of pharmacological interest [11]. After a first report on inhibition of human cholinesterases (hChEs) by a family of ^18^F-acetophenones developed for PET neuroimaging [12], it was of interest to investigate thoroughly the carbon analogue of Zifrosilone (Figure 1B), 1-(3-*tert*-butylphenyl)-2,2,2-trifluoroethanone (TFK) (Figure 1C), to describe the mechanism of inhibition of hAChE by kinetics and molecular modeling approaches. Moreover, possible modulation and/or protection of AChE by TFK against OP phosphylation was explored.

## 2. Materials and Methods

### 2.1. Chemicals

TFK was synthesized as described in [12]. Solution of TFK (0.1 M) was made in acetonitrile. Echothiophate iodide was from Biobasal AG (Basel, Switzerland). Stock solution of echothiophate (0.1 M) was made in water. Paraoxon was purchased from Sigma-Aldrich (Saint Louis, MO, USA). Stock solution of paraoxon (0.1 M) was made in EtOH. Cresylsaligenyl phosphate (CSP) was a gift from Prof O. Lockridge (UNMC, Omaha, NE, USA). Solution of CSP (0.1 M) was made in acetonitrile. Acetylthiocholine iodide was from Sigma-Aldrich (Saint Louis, MO, USA). Stock solution of ATC (0.1 M) was made in water. Stock solutions of substrate and inhibitors were stored at −20 °C.

Dithiobisnitrobenzoic acid (DTNB) was from Sigma-Aldrich (Saint Louis, MO, USA). 50 mM solution of DTNB was prepared as described in Ellman [13]. Calbiochem Probe IV (3-(7-Hydroxy-2-oxo-2H-chromen-3-ylcarbamoyl) acrylic acid methylester) was from Merck Millipore (Darmstadt, Germany). Stock solution of Probe IV (1 mM) was in DMSO and stored at −20 °C. All other chemicals were of biochemical grade.

### 2.2. Enzymes

Recombinant human AChE monomer (MW = 70,000 Da) was expressed in CHO-K1 (Chinese-hamster ovary, ATCC) cells [14]. AChE purification was carried out as described [15] The enzyme was concentrated to 14.7 mg/mL using a Vivaspin 6 (30,000 MW cutoff, Sartorius) and dialyzed at 4 °C against 10 mM HEPES pH 7.5 buffer containing 10 mM NaCl. The active site concentration of AChE was determined by the method of residual activity according to Leuzinger [16], using echothiophate, as titrating agent. The residual activity after complete phosphorylation by each concentration of echothiophate was measured by the Ellman’s method [13] in 0.1 M sodium phosphate, pH 8.0 at 25 °C with 1 mM ATC as the substrate. The active site concentration of the pure rhuAChE monomer was 2.1 × 10^−4^ M. The working enzyme was diluted 20,000 time in 0.1 M phosphate buffer containing 1 mg/mL BSA ([E]_w_ = 1 × 10^−8^ M).

### 2.3. Kinetic Study of Inhibition

Kinetic studies of human recombinant AChE inhibition by TFK were performed in 0.1 M sodium phosphate buffer pH 8.0 at 25 °C, using the Ellman’s method [13] with acetylthiocholine (ATC) as the substrate. Three different concentrations of ATC were used (0.1, 0.5 and 1 mM): Assays were initiated by addition of the enzyme. The final enzyme concentration, E, was 1 × 10^−10^ M.

After rapid mixing of solutions, absorbance change 412 nm was recorded for 60 min using a spectrophotometer PerkinElmer λ25 with photodiode detector (PerkinElmer Inc., Waltham, MA, USA). The inhibition process was characterized by a slow onset before reaching steady state. This process is described by Equation (1):

(1) $${\left[P\right]}_{t}=v_{\mathrm{ss}}t+\frac{\left(v_{i}-v_{\mathrm{ss}}\right)\left(1-\exp\left(-k_{\mathrm{obs}}t\right)\right)}{k_{\mathrm{obs}}}$$

where *v_i_* is the initial velocity, *v_ss_* the steady-state velocity and *k_obs_*, the first-order rate constant associated with establishment of the steady state. The reciprocal of *k_obs_* is the lag time before steady state. This is characteristic of slow binding inhibition [17,18,19].

The initial reversible inhibition step was analyzed from the tangents of progress curves. The general case for linear reversible inhibition is described by following rate Equation (2):

(2) $$v_{i}=\frac{V_{max}\left[S\right]}{K_{m}\left[1+\left(\left[I\right]/K_{ci}\right)\right]+\left[S\right]\left(1+\left(\left[I\right]/K_{ui}\right)\right)}$$

where *V_max_* is the maximum velocity, [*S*] the substrate concentration, *K_m_* the Michaelis-Menten constant, [*I*], the inhibitor concentration, *K_ci_*, the competitive inhibition constant, and *K_ui_*, the uncompetitive inhibition constant. Rearranging Equation (2) as 1/*v_i_* or [*S*]/*v_i_* as a function of [*I*] provides two complementary plots as decribed by Cornish-Bowden [20]. In the limiting case of competitive inhibition, *K_ui_*→∝, while for uncompetitive inhibition, *K_ci_*→∝. Non-competitive inhibition gives *K_ci_* = *K_ui_* and mixed-type inhibition gives *K_ci_* ≠ *K_ui_*. Therefore, inhibition constant and type of reversible inhibition of the fast step were determined according to the graphical methods of Cornish-Bowden [20] by building Dixon plots (1/*v_i_* vs. [TFK]) and Cornish-Bowden plots ([ATC]/*v_i_* vs. [TFK]) at 3 different ATC concentrations [*S*] = 0.1; 0.5 and 1 mM). 

Diagnosis of type of SBI and binding kinetic parameters and inhibition constant corresponding to final reversible step where determined from secondary plots, *k_obs_* vs. [TFK] at 3 different ATC concentration [5].

For the study of transient enzyme acylation by 2 × 10^−8^ M TFK and slow reactivation, the enzyme activity was monitored by the Ellman assay [13]. However, for analyzing slow reactivation after 10- and 1000-fold dilution of the system, because the enzyme concentration became very low (10^−10^ and 10^−12^ M), the recovery of activity was monitored at 25 °C under the same buffer conditions by high sensitivity spectrofluorimetric assay, using the Calbiochem thiol Probe IV [21] instead of DTNB. The Calbiochem probe IV is a coumarinyl derivative that reacts with thiol-chemicals to form a highly fluorescent conjugate. It was found to be the fastest and the most sensitive thiol reagent. Assay of ChEs using this thiol reagent instead of DTNB is more than 2 orders more sensitive than the classical Ellman assay, allowing measurement of activity in media containing <10^−11^ M ChE. Kinetic and molecular modeling studies showed that Probe IV does not interfere with activity measurements so that ATC-based assays using DTNB and Probe IV are correlated. The experimental conditions of assay in a Peltier thermostated spectrofluorimeter F-7100 (Hitachi Ltd., Tokyo, Japan) were previously decribed [21,22].

### 2.4. Modulation of AChE Phosphorylation Following Enzyme Preincubation in the Presence of TFK

Two OPs were selected, CSP and paraoxon. Inhibition of hAChE by CSP (0.21 µM) was performed in 0.1 M phosphate buffer, pH 8.0 after different pre-incubation times ranging from 5 to 120 min in the presence of various concentrations of TFK (0.1–10 nM). We investigated the effect of pre-incubation by TFK (1–10 nM) on inhibition of huAChE by 50 nM paraoxon under the same conditions.

### 2.5. Molecular Modeling

#### 2.5.1. Molecular Docking

Molecular docking was performed using as targets several structures of hAChE co-crystallized with different non-covalent inhibitors (PDB ID 4EY4-4EY8 [15]) and one covalently bound to an organophosphorus adduct (human AChE phosphonylated by sarin, PDB ID 5FPQ, [23]). These structures are missing peripheral loop fragments 259–264 and 495–497. These were inserted from another hAChE X-ray structure (PDB ID 4BDT [24]). Inhibitors, ions and water molecules were removed prior docking. Hydrogen atoms were added with respect of hydrogen bonding network by Reduce software [25]. Molecular docking with a Lamarckian Genetic Algorithm (LGA) [26], was performed with Autodock 4.2.6 [27] software. Grid box for docking of 30 Å × 30 Å × 30 Å included the whole gorge from the mouth to the active site, including PAS. In spite of low number of torsion degrees of freedom of the inhibitor, number of docking runs was increased to analyze cauterization (2 Å tolerance). The main of selected LGA parameters were as follows: 2000 runs, 25 × 10^6^ evaluations, 27 × 10^4^ generations and population size 300. 

#### 2.5.2. Molecular Dynamics

For the preparation of the model systems and further analysis of MD trajectories, VMD software [28] was used.

*Ligand parameterization*: For TFK and TMTFA molecules parameters were taken from Charmm General Force Field [29] through CGenFF interface (https://cgenff.umaryland.edu/), missing parameters were adjusted with the help of *ff*TK plugin of VMD [30,31]. 

*System setup*: For MD simulations, complexes of hAChE with the inhibitors (TFK and TMTFA) obtained from molecular docking (X-ray structure PDB ID 4EY7 as a target, binding pose with inhibitors in the active site) were chosen. TIP3P water box was added with boundaries in at least 10 Å from protein and ligand atoms. Sodium and chloride ions were added to final concentration of 0.15 M. Final size of both systems was 67,804 atoms, 84.6 Å × 85.8 Å × 95.4 Å. 

For all MD simulations, NAMD 2.11 software [32] with CHARMM36 force field [33] was used in NPT ensemble (298 K, 1 atm) with periodical boundary conditions. MD simulations were run at the Lomonosov Moscow State University supercomputer [34]. 

For pre-optimization, coordinates of all atoms present in X-ray structure were fixed. Positions of all added atoms were minimized in 5000 steps and subjected 5 ns MD simulation to optimize water box and added loops. Then, all atoms were minimized in 5000 steps. This structure was used as reference and starting point for targeted molecular dynamics (TMD) and QM/MM calculations.

To create starting points for umbrella-sampling free energy calculations, pathways of pulling the inhibitors inside hAChE active site gorge and outside the protein, were obtained by means of targeted molecular dynamics with initial structure as a reference (see Supplementary Materials for details).

*Umbrella sampling (US)*: Pathways obtained by TMD were divided into 240 windows separated by 0.25 Å. State in each window was sampled during 1 ns simulations with harmonic restraining force 50 kcal/mol·Å^2^, with RMSD of trifluoroacetophenone part of the inhibitors as a collective variable. These parameters were adjusted in series of test runs and provided good overlapping of histograms. To construct PMF weighted histogram analysis method (WHAM) [35,36] in A. Grossfield implementation, v. 2.0.9 (http://membrane.urmc.rochester.edu/content/wham) was used.

*Replica exchange molecular dynamics (REMD-US)*: To ensure better sampling, after analysis of PMF obtained by US, 168 of US windows were used as initial coordinates of REMD-US replicas [37,38] with the same other parameters. All replicas were simulated concurrently having Hamiltonians with different biasing potentials. Every 100 steps replicas of the system underwent exchange performed using the Metropolis Monte Carlo criterion. Simulations were performed until full convergence during 10 ns for each replica.

#### 2.5.3. QM/MM Calculations

Initial systems of hAChE-TFK/TMTFA complexes were taken after MD optimization. First solvation shell (water molecules within 2 Å from the protein) was kept. QM/MM calculations were performed with the NwChem 6.6 software [39]. The density functional theory approach with Grimme empirical dispersion correction [40] PBE0-D3/cc-pvdz was used in QM part. The MM subsystem was modeled with the AMBER force field [41]. Quantum sub-system includes the whole inhibitor molecule and active site residues: the catalytic triad Ser203, His447, Glu334; oxyanion hole Gly121, Gly122, Ala204; other principal residues forming hydrogen-bonding network around the catalytic residues, Glu202, Ser229, Glu450 and three water molecules between them, 129 atoms total, including link atoms. Total size of the system was ~11,500 atoms. For the reactivation process, distance between the inhibitor carbonyl atom and Ser203 O^γ^ atoms was increased using harmonic constraints. Unconstrained optimization was performed for obtained reactivation pathway points.

## 3. Results and Discussion

### 3.1. Slow-Binding Inhibition Kinetics of rhAChE by TFK

**Figure 2:**
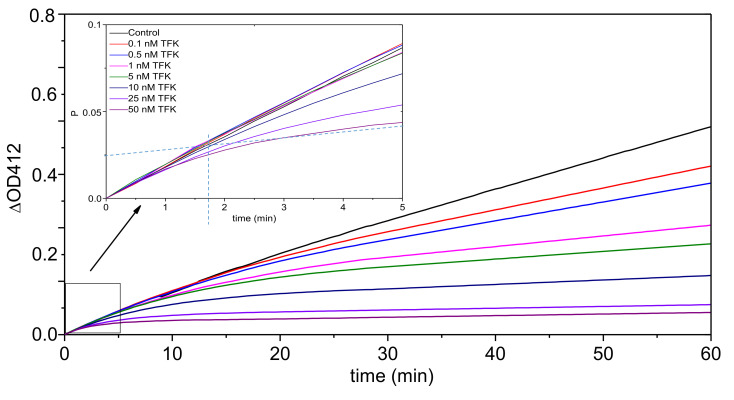
Typical progress curves (without treatment for smoothing noise) for slow-binding inhibition of AChE by TFK. [E] = 0.08 nM, [ATC] = 0.1 mM and [TFK] ranged from 0.1 to 50 nM. ΔOD_412_ is the concentration of ATC hydrolysis product (in Equation (1), [P] = thiocholine = thionitrobenzoate), expressed as the absorbance increase at 412 nm. The reciprocal of *k_obs_* is the lag time (vertical dotted line at *t* = 1.75 min for 50 nM TFK) before steady state. Progress curves fit to Equation (1).

Inhibition kinetics of hAChE by TFK ranging from 0.1 to 50 nM was recorded for 60 min in the presence of ATC (0.1, 0.5 and 1 mM). Kinetic analysis of inhibition progress curves showed that there is a slow onset of inhibition before reaching the equilibrium in less than 4 min (Figure 2). This is in agreement with what was previously reported [12]. The steady state was established in less than 35 min. The dependence of the first order rate constant (*k_obs_*), the reciprocal of the lag time, of the pre-steady state phase on TFK concentration was analyzed according to the formalism of slow binding inhibition (SBI) [5]. 

**Figure 3:**
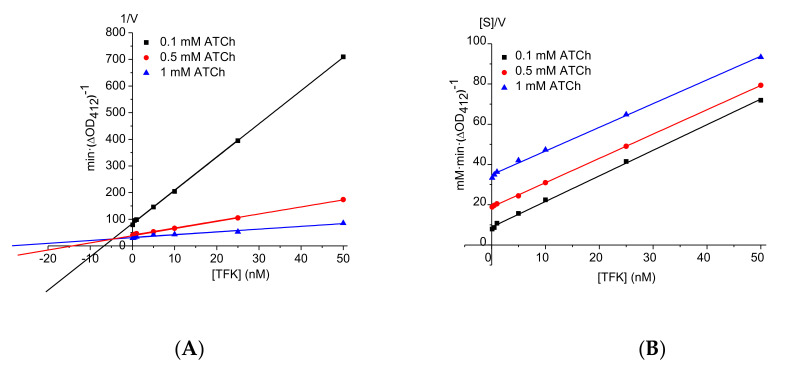
Dixon plot (**A**) and Cornish-Bowden plot (**B**) for determination of fast reversible inhibition constant.

Initial fast inhibition type and inhibition constant (*K_i_* = *k*_−3_/*k*_+3_) for the first binding step (formation of EI) were determined from tangents to progress curves *k_obs_* vs. time (Figure 2), Dixon and Cornish-Bowden plots (Figure 3). Three different concentrations of ATC were used (0.1; 0.5 and 1 mM). Panels A and B in Figure 3 provide evidence that fast reversible inhibition step is competitive (Dixon plots are intersecting at-[TFK] = *K_ci_* and Cornish-Bowden plots are parallel) with *K_ci_* = *K_i_* = 5.15 ± 0.36 nM.

**Figure 4:**
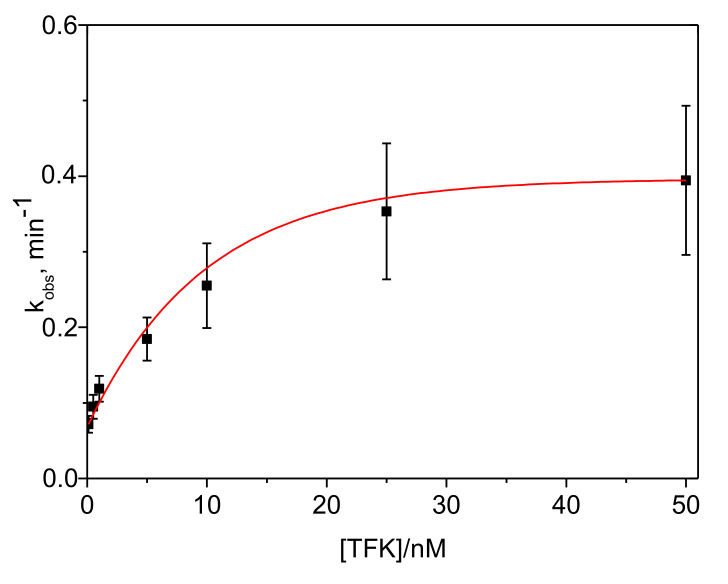
Typical dependance of *k_obs_* as a function of TFK concentration for inhibition in the presence of 0.1 mM ATC. The *k_obs_* values were determined from nonlinear fitting of progress curves in Figure 2 at three substrate concentrations. Data were fitted to Equation (3): ordinate is *k*_−4_ and asymptote is *k*_−4_ + *k*_+4_ in Scheme of Figure 5.

The plots of *k_obs_* vs. [TFK] were built for each ATC concentration (Figure 4). This diagnosis plot established the type of SBI.

**Figure 5:**
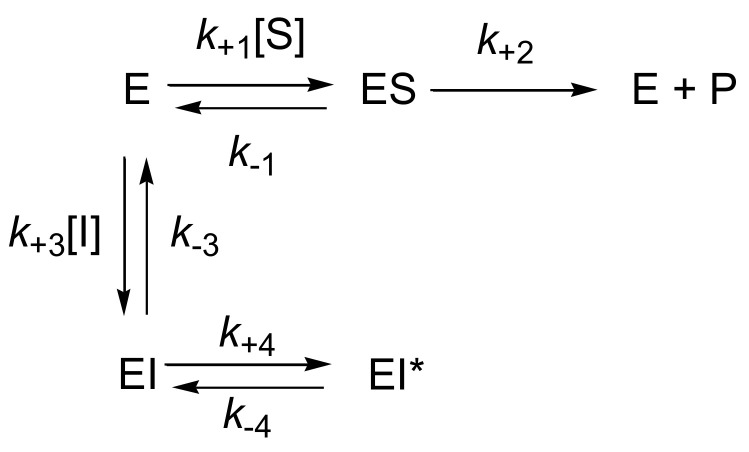
Slow-binding inhibition model of type B.

Plots of *k_obs_* vs. [TFK] are upward hyperboles (Figure 4). This is characteristic of SBI of type B (Figure 5). Equation (3) describes the dependence of *k_obs_* on inhibitor concentration, [*I*], in SBI of type B.

(3) $$k_{obs}=k_{-4}+\frac{k_{+4}\left[I\right]}{K_{I}\left(1+\left[S\right]/K_{M}\right)+\left[I\right]}$$

Accordingly, after rapid formation of a first complex EI, characterized by an inhibition constant *K_i_*, the enzyme undergoes a slow «isomerization», leading to a second complex EI* characterized by a stronger ligand binding affinity (K_i_* < *K_i_*).

The binding kinetic parameters were determined from non-linear curve fitting of secondary plots *k_obs_* vs. [TFK] (Figure 4) obtained for inhibition in the presence of 0.1, 0.5 and 1 mM ATC. In Figure 4 the ordinate intercept is *k_−4_* and the asymptote is *k*_−4_ + *k*_+4_. The constants *k*_−4_ and *k*_+4_ were found to be independent on ATC concentration, as expected for SBI of type B. Thus, their averages values are *k*_−4_ = 0.054 ± 0.006 min^−1^ and *k*_+4_ = 0.456 ± 0.095 min^−1^. Then, the inhibition constant corresponding to formation of EI*, K^*^_i_ = 0.53 nM, was determined as follows: K^*^_i_ = *K_i_* *k*_−4_/(*k*_−4_ + *k*_+4_) = 0.53 ± 0.07 nM. This value is in agreement with values previously determined by two different methods (0.4 and 0.56 nM) in the presence of 5 mM ATC in 0.1 phosphate buffer pH 7.4 after 70 min incubation at 23 °C [12].

Other important binding kinetic parameters are the residence time on AChE (the reciprocal of the overall *k_off_* rate constant, *τ* = 1/koff) and the fractional occupancy of AChE (*FO_t_*) [42]. These parameters were derived from elementary kinetic constants (Equations (4) and (5)):

(4) $$\tau =\frac{\left(k_{-3}+k_{+4}+k_{-4}\right)}{k_{-3}k_{-4}}$$

(5) $$FO_{t}=\frac{{\left[I\right]}_{t}}{{\left[I\right]}_{t}+(k_{-3}/\left(k_{+3}+(k_{+3}k_{+4}\right)/k_{-4})}$$

However, as seen in Equations (4) and (5), estimation of *τ* and *FO_t_* implies knowledge of *k*_−3_, and *k_on_*, the second-order rate constant for the initial binding step to hAChE. This constant is not known for TFK in hAChE. However, apparent values of *k_on_* of several trifluoroketones for binding to different AChE were reported. These constants were determined assuming a single binding step process. For binding of neutral aliphatic trifluoroketones to electric eel AChE, *k_on_* = 1–5 × 10^9^ M^−1^ min^−1^ [2]. For binding of TFK to mouse AChE, *k*_on_ was similar, 3 × 10^9^ M^−1^ min^−1^ [43]. On the other hand, for the charged counterpart of TFK, TMTFA (Figure 1), an SBI of type A (a single slow step corresponding to formation of EI), *k_on_* is slower, 6 × 10^6^ M^−1^ min^−1^ for electric eel AChE [3], while for the neutral silylated homologue, Zifrosilone (Figure 1), *k_on_* = 6 × 10^6^ M^−1^ min^−1^ for electric eel AChE [7]. The *k_on_* values for binding of neutral trifluoroketones, are of the order of the values reported for binding of small drugs to biological targets, 6 × 10^7^ to 6 × 10^9^ M^−1^ min^−1^ [44]. Thus, it may be assumed that the second order association rate constant of TFK in hAChE (*k*_+3_) is close to the *k_on_* value for mouse AChE. Thus, taking *k*_+3_ ≈ 3 × 10^9^ M^−1^ min^−1^, with 〈*K_i_*〉 = 5.15 nM, it follows that *k*_−3_ ≈ 15.45 min^−1^. This leads to an overall *k_off_* ≈ 0.052 min^−1^ and *τ* = 19 ± 2 min. The half-time for dissociation of reversibly bound TFK is t_1/2_ = ln2/*k_off_* ≈ 13 min. The silylated homologue displays a much longer residence time of about 70 h for rat brain AChE, although its affinity is close to that of TFK (*K_i_* = 0.26 nM) [7].

Because both the concentration of drug in the target compartment and the residence time on physiological target as a function of time, determine the duration of action of a drug in the body, the pharmacological efficacy of a drug depends on the fractional occupancy of the enzyme as a function of time (*FO_t_*) [42,44]. Then, *FO_t_* depends on both the pharmacokinetic profile of considered drug, i.e., its concentration in the central compartment (the blood circulation) as a function of time, and the binding kinetic parameters on target(s). *FO_t_* change with time is therefore a useful theoretical parameter for estimating the potential pharmacological interest of drugs with long residence times on the target. For TFK, as a SBI of type B, *FO_t_*, for different [I]_t_ can be calculated from Eqation (5). Assuming that the initial concentration of TFK in blood is 4.3 μM at time 0 (e.g., after intravenous injection to mice of a dose of 1 mg/kg), it follows that *FO_t_*_0_ = 99.9%. After a certain time (*t*’) when concentration in blood has dropped to 4.3 nM, *FO_t_*_0_ = 88.7%. If after time *t*”, the concentration has decreased by another order of magnitude (4.3 pM), then *FO_t_*_0_ = 7.3%. Thus, it is clear that with a residence time on AChE of about 20 min, the potential pharmacological efficacy of TFK could be maintained at a high level even though the drug concentration in blood has decreased to a very low value.

### 3.2. Transient Acylation of AChE by TFK and Subsequent Enzyme Reactivation

Binding of a transition state analogue, like acetophenone, to ChEs, the formation of a transient hemiketal conjugate mimicking the acetyl tetrahedral intermediate was observed or hypothesized in some cases [6,43]. Thus experiments were performed to study enzyme spontaneous reactivation.

**Figure 6:**
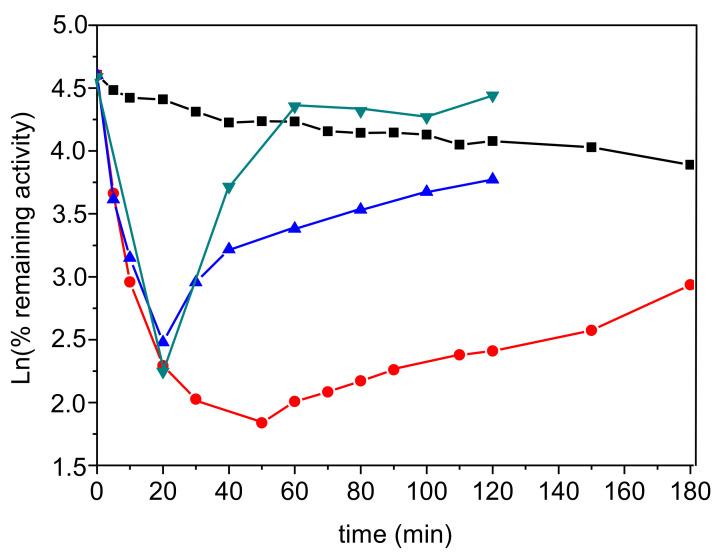
SBI and subsequent acylation of 2 × 10^−9^ M AChE by 2 × 10^−8^ M TFK, followed by slow deacylation. Activity was assayed with 1 mM ATC with DTNB as the thiol probe (black, blue, red curves) and with Probe IV as the thiol probe (green curve). The black curve is enzyme activity (control) in the presence of 2% acetonitrile; the red curve is enzyme activity monitored up to 180 min in the presence of 2 × 10^−8^ M TFK. Blue curve: after steady-state SBI, the system was diluted 10 times, so that TFK dropped to 2 × 10^−9^ M after *t* = 20 min. Green curve: after steady-state SBI, the system was diluted 1000 times, then TFK final concentration dropped to 2 × 10^−11^ M after *t* = 20 min. The first-order rate constant of deacylation (*k_reac_*) was estimated from the slope of Ln increase in activity vs. time.

The reaction process was performed under pseudo-first order conditions to reach complete inhibition of enzyme by 2 × 10^−8^ M TFK. Then, the enzyme activity was monitored up to 180 min, far beyond establishment of reversible steady-state equilibrium. During this second step, the recovery of enzyme activity was monitored without dilution or after dilution in order to drop TFK concentration in the medium. As seen in Figure 6 red curve, after formation of the second reversible complex EI*, the enzyme activity progressively started to increase. This suggested, that after formation of EI* the enzyme was transiently acylated, and then slowly deacylated. However, during the putative deacylation process, the enzyme could have been re-inhibited by the excess of TFK present in the medium. Thus, after 20 min inhibition, the system was diluted 10- and 1000-fold to drop TFK concentration, and the enzyme activity was monitored as a function of time. Because after dilution, the enzyme activity became very low for accurate monitoring of activity vs. time, instead of the classical Ellman assay, the activity was monitored using a new method with the fluorescent thiol probe (Calbiochem Probe IV) instead of DTNB [21,22]. Results showed that dilution of TFK speeded up the deacylation process (blue and green curves in Figure 6, showing faster recovery of enzyme activity). In the presence of non-inhibitory TFK concentration (2 × 10^−11^ M), enzyme reactivation was completed in 40 min with *k_react_* ≈ 0.05 min^−1^; t_1/2_ ≈ 14 min (Figure 6, green curve) instead of more than 3 h in the presence of 2 × 10^−8^ M TFK (Figure 6, red curve).

The slow enzyme reactivation process was interpreted as spontaneous slow deacylation of bound TFK from AChE active center. Molecular modeling (see next section) confirmed the occurrence of transient acylation of AChE active site serine after TFK binding and shed light on molecular interactions involved in these acylation and deacylation steps. Then, considering that the residence time (*τ*) on AChE calculated from reversible SBI is 20 min, the subsequent acylation and deacylation processes provide an overall residence time of about 60 min.

The slow recovery of enzyme activity depends on the TFK concentration present in the medium. In the presence of remaining 0.02 nM TFK (green curve), it takes less than 40 min to regain an activity similar to that of control enzyme at *t* = 60 min. 

### 3.3. Molecular Modeling of Interaction between TFK and AChE

Molecular modeling was used to depict in terms of molecular events the different steps of TFK interactions with huAChE. Preliminary results about interaction between TFK and AChE were recently reported [45]. 

In majority of docking results, TFK was found in the peripheral anionic site (PAS) or bottleneck area (Figure S1). The dominating poses (red clusters in Figure S1) were stabilized by C-Hal…π interactions of –CF_3_ group with Trp286 ring (Figure S2A). In another pose within cluster making up the red structures, the trifluoro moiety produces hydrogen bonds with the peptide backbone of Phe295, Arg296 (Figure S2B) in a way, similar to interactions in the oxyanion hole. In the next less populated clusters (orange and yellow structures) there were other poses with the same binding pattern (Figure S2C), showing interactions with Ser293 peptide backbone and possible hydrogen bond between the trifluoro moiety and side chain hydroxyl group (Figure S2D). In general in PAS, trifluoroketo-group interacts with the acyl-binding loop as with a huge oxyanion hole, trapping it near entrance to the gorge. Among complexes of AChEs with inhibitors, containing a trifluoro moiety, the majority of them are structures of non-hydrolysable substrate analogues covalently bound to the catalytic serine, and will be discussed below. However, one X-ray structure shows a non-covalent inhibitor, N-(2-diethylamino-ethyl)-3-trifluoromethyl-benzenesulfonamide (PDB ID: 4B84, [46]) with the trifluoro moiety bound in the PAS in the similar fashion (Figure S3), oriented by interactions with acyl-loop main chain NH group.

Binding poses of TFK suitable for covalent interactions with the catalytic triad were found only for X-ray structures PDB ID: 4EY7 and PDB ID: 5FPQ as targets in minor clusters. 4EY7 co-crystallized with donepezil has a wider gorge than the other X-ray structures due to rotation of Tyr337. In many cases this allows to better accommodate bulky inhibitors [47]. In this case, rotation of the catalytic serine side chain, forming hydrogen bond with Glu202, not His447 as usual, gives space for TFK to enter in the active site. Though this is not enough, carbonyl oxygen atom is slightly displaced from the oxyanion hole in these cases, indicating that Trp86 side chain has to move a bit for better fit. Following QM/MM optimization provided necessary expansion of the active site and full accommodation of the inhibitor. X-ray structure 5FPQ is the non-aged covalent conjugate of AChE with sarin. The methyl isopropoxy phosphonyl adduct expands the active site. In particular, Trp86 side chain is moved a bit (Figure S4). These displacements provide enough space for TFK, though between two possible poses, one has *tert*-butyl moiety outside cation-binding site Trp86 and Glu202 (Figure S5A, PDB ID: 4EY7 as a target), and in the other pose, the phenyl ring is out of plane of the trifluoroketo-group (Figure S5B, PDB ID: 5FPQ as a target).

The TFK pose at the bottom of the gorge but outside the active site (Figure S5C) is interesting. Indeed, such a pose was observed only in docking with X-ray structure PDB ID: 4EY8 (complex between AChE and Fasciculin-2) as a target. This provides an additional evidence for Fasciculin-induced conformational changes of AChE, widely discussed previously [48,49].

**Figure 7:**
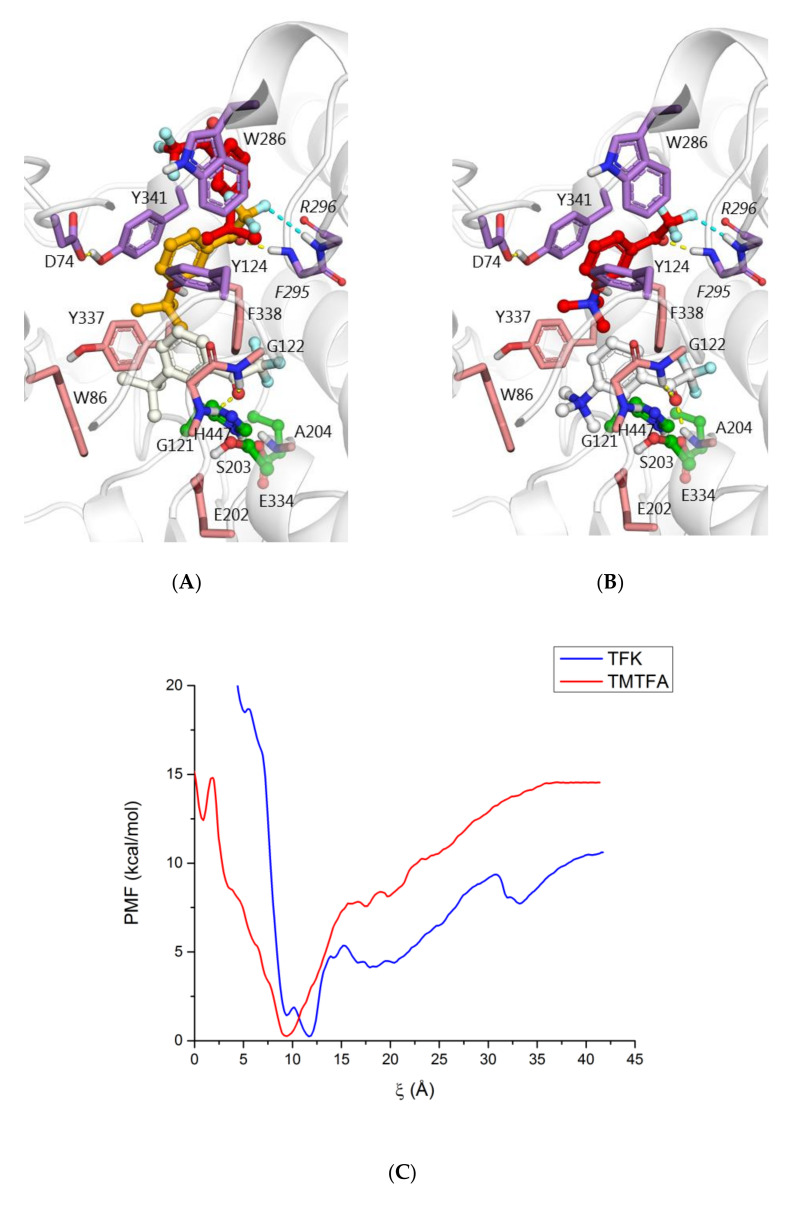
Binding of TFK to AChE: (**A**) main docked poses in the gorge: in the PAS (most populated cluster, carbon atoms colored red), at the gorge rim partly blocking the bottleneck (carbon atoms orange), and in the active site ready to react with the catalytic serine (least populated cluster, carbon atoms are colored white) compared to the main binding poses of TMTFA (**B**). Docking results with X-ray structure PDB ID: 4EY7 as a target are shown. (**C**) Free energy (PMF) profile of the binding process calculated with REMD-US method. Process coordinate ξ is the distance between the TFK/TMTFA trifluoroketone group and the active site, oxyanion hole and catalytic serine hydroxyl-group.

Generalizing docking results, 3 major binding poses can be outlined: in the PAS, at the area of bottleneck and in the active site (Figure 7A). For comparison, docking results for TMTFA were less diverse, and binding at the area of bottleneck was dominating, while binding to the PAS was negligible. Binding to the active site is similar to TFK, with the trimethyl ammonium group slightly closer to Glu202 due to electrostatic attraction (Figure 7B).

Potential of mean force (PMF) profiles for binding of TFK and TMTFA to hAChE calculated using REMD-US method are in agreement with docking results (Figure 7C). For TFK, two close minima at level 9 and 12 Å, corresponding to binding in the PAS and bottleneck are seen, while for TMTFA only one global minimum at level 9 Å, corresponding to binding in the area of the bottleneck was observed. Transition from these favorable positions in the middle of the gorge to the active site is associated with energy barriers. For TMTFA of 15 kcal/mol, and for TFK it is much higher, more than 20 kcal/mol. 

In addition to the two distinctive positions inside the gorge, PMF for TFK allows to assume non-specific interactions on the protein surface (local minima valleys in area 17–18 Å and 33–34 Å from the gorge bottom, Figure S6), additionally slowing down the inhibition process. In contrast, TMTFA slides directly into the gorge. This is an illustration of the effect of the positive charge and the role of AChE as a macro-dipole [50,51]. This electrostatic effect acts as a driving force for trafficking of the inhibitor down the gorge and its incorporation in the active site, as it was previously discussed [51,52,53,54].

**Figure 8:**
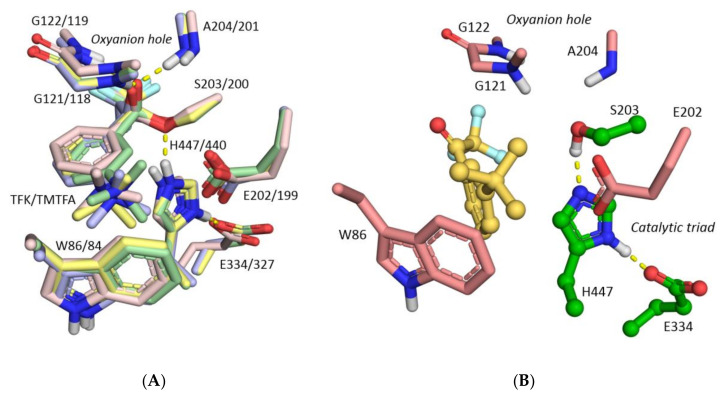
(**A**) Tetrahedral adducts for reaction between TFK (carbon atoms shown in yellow) and TMTFA (carbon atoms shown in green) and hAChE obtained by QM/MM calculations overlaid with X-ray structures of conjugates of TMTFA with *Mus musculus* (PDB ID: 2H9Y [53], carbon atoms shown in blue) and *Torpedo californica* (PDB ID: 1AMN [6], carbon atoms shown in pink). Double hAChE/TcAChE numbering is provided; (**B**) Non-covalent complex between TFK and hAChE, stable product resulting from the tetrahedral adduct reactivation process.

QM/MM optimization of non-covalent complex of hAChE with TFK in the active site obtained by molecular docking leads to size expansion of the active site necessary to accommodate the inhibitor and lower energy barrier for formation of covalent adduct. Traditionally, resulting hemiketals are called “transition state analogs”, though practically they are rather analogs of tetrahedral intermediate of AChE-catalyzed ester hydrolysis [55,56]. Hemiketal configuration is very close for TFK and TMTFA, with the only difference that trimethyl ammonium group of TMTFA is 0.6 Å closer to Glu202 than *tert*-butyl moiety of TMTFA. Accordingly, it is similar to configuration X-ray structure of TMTFA bound to *Mus musculus* (PDB ID: 2H9Y [53]) and *Torpedo californica* (PDB ID: 1AMN [6]) AChE (Figure 8A). 

Energy scan for reactivation process shows that while inhibitor is inside the active site, i.e., carbonyl oxygen atom is within the oxyanion hole, formation of the tetrahedral adduct occurs spontaneously. Stable noncovalent complex was obtained only when the TFK group left the oxyanion hole (Figure 8B). 

Though reaction itself occurs barrier-less, it has associated energy barrier for incorporation of the TFK group into the oxyanion hole, estimated with QM/MM method as 19.3 kcal/mol for TFK and 13.0 kcal/mol for TMTFA. Energy barrier for TMTFA estimated with QM/MM method corresponds to values obtained with REMD-US (Figure 7C, free energy profile, 15 kcal/mol energy barrier between position in the gorge at 9 Å and local minima at 1 Å, corresponding to position in the active site), while for TFK, barrier calculated with classical methods was overestimated, probably due to lack of polarizability in classical molecular dynamics.

Molecular modeling results suggest that binding of TFK to hAChE is slow due to non-specific interactions on the protein surface, multiple binding poses inside the gorge (EI*), and high energy barrier associated with induced-fit entrance of the inhibitor into the active site. Absence of positive charge additionally slows down this process compared to positively charged analogs. 

### 3.4. Modulation of AChE Phosphylation by Pre-Incubation with TFK

**Figure 9:**
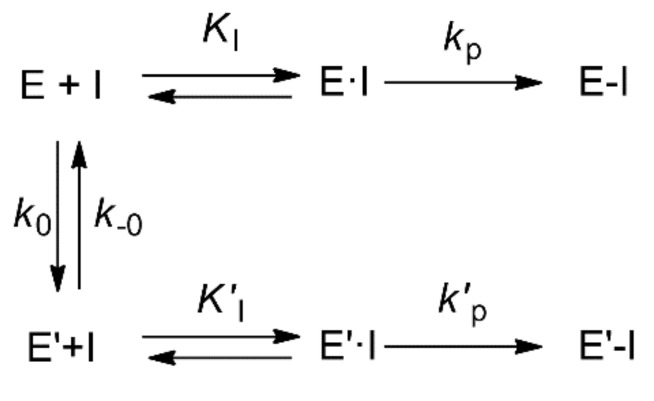
Slow-binding inhibition of type C with a subsequent covalent step, enzyme phosphorylation (*k_p_*) in the present case.

Then, we investigated the possible modulating or protecting effect of TFK on AChE phosphylation by two different OPs. The first OP was cresylsaligenyl phosphate (CSP), the active metabolite of tricresylphosphate. CSP is a strong phosphorylating agent of ChEs [57,58]. Inhibition of ChEs by CSP under first order conditions is biphasic. This atypical phosphylation process has been reported with other OPs and carbamates [45], and was interpreted it in terms of SBI of type C. Accordingly, the enzyme exists as two forms, E and E’ in slow equilibrium, each form reacting at different rates with the agent I (Equations (6)–(9) and Figure 9) [57,58].

(6) $$\left[E_{\mathrm{tot}}\right]=\left[E_{0}\right]+\left[E_{0}^{\prime }\right]$$

(7) $$\left[E_{t}\right]=\left[E_{0}\right]\exp\left(-k_{\mathrm{obs}}t\right)+\left[E_{0}^{\prime }\right]\exp\left(-{k^{\prime }}_{\mathrm{obs}}t\right)$$

(8) $$k_{\mathrm{obs}}=\frac{k_{p}\left[I\right]}{K_{I}+\left[I\right]}$$

(9) $${k^{\prime }}_{\mathrm{obs}}=\frac{{k^{\prime }}_{p}\left[I\right]}{{K^{\prime }}_{I}+\left[I\right]}$$

This type of SBI followed by a covalent step, i.e., phosphylation, is related to enzyme hysteresis [59]. These mechanisms have been thoroughly investigated [60,61]. Thus, we hypothesized that TFK could affect the phosphorylation process by preferential binding to one or both enzyme forms.

Inhibition of hAChE by CSP (0.21 µM) performed after different pre-incubation times in the presence of various concentrations of TFK (0.1–1 nM) showed that the biphasic progressive inhibition process of the enzyme is affected by TFK. The biphasic progressive inhibition was analyzed as a function of pre-incubation time and concentration in TFK.

**Figure 10:**
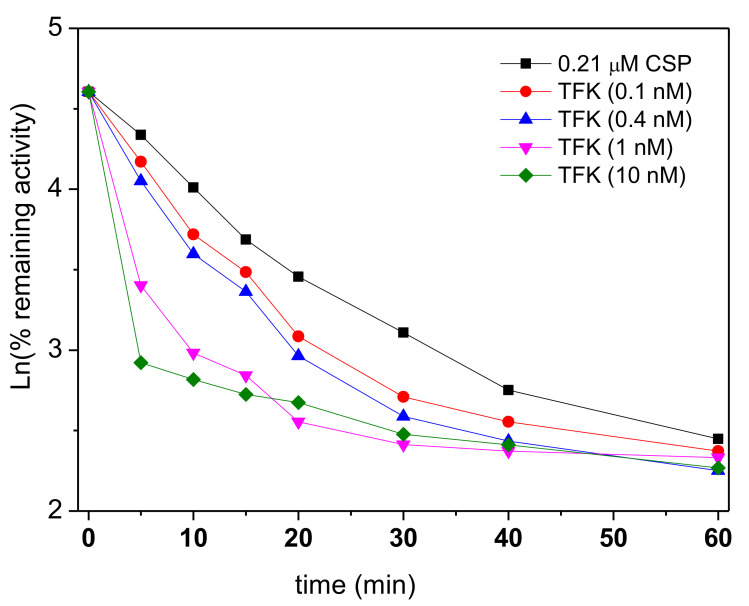
Modulation of progressive biphasic inhibition of hAChE by CBDP CSP (0.21 µM) after pre-incubation of the enzyme for 120 min in the presence of TFK (0.1–10 nM).

**Figure 11:**
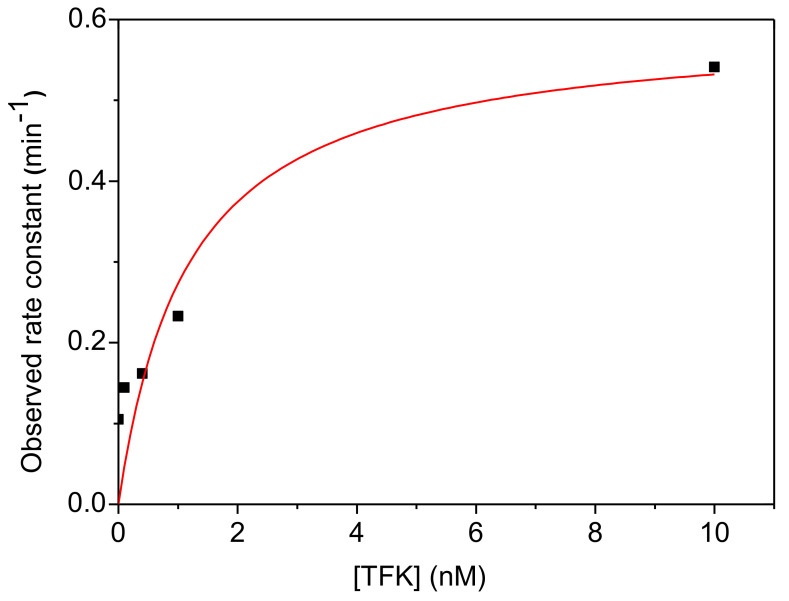
Dependence of *k*_obs,max_ (fast process, see Equations (7) and (8)) of hAChE phosphorylation by CSP as a function of TFK concentration (after 120 min pre-incubation).

**Figure 12:**
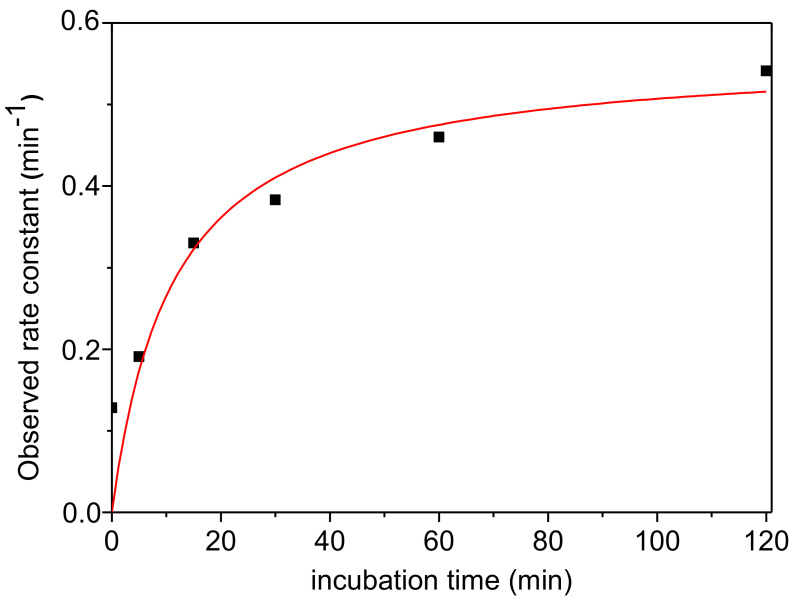
Observed first process (fast process) phosphorylation rate constant of hAChE by 0.21 μM CSP as a function of enzyme pre-incubation time in the presence of 10 mM TFK.

It was found that pre-incubation of AChE with TFK increased the observed rate of the fast process of phosphorylation but has no significant effect on the slow process (Figure 10). The effects of pre-incubation duration and TFK concentration on the rate of inhibition was directly quantified from the slope of *Ln* (residual Activity) vs time of the fast phosphorylation process and pre-incubation duration the residual activity increased as pre-incubation duration and TFK concentration increased. The deleterious effect of TFK was maximum for 10 nM TFK (Figure 11) and reached a plateau after 120 min pre-incubation (Figure 12).

Because of the hysteretic behavior of AChE with CSP as a phosphorylating agent, the modulating effect of enzyme pre-incubation in the presence of TFK, makes the enzyme form E more susceptible to phosphorylation by CSP, and accelerates the overall phosphorylation rate of the enzyme system. 

**Figure 13:**
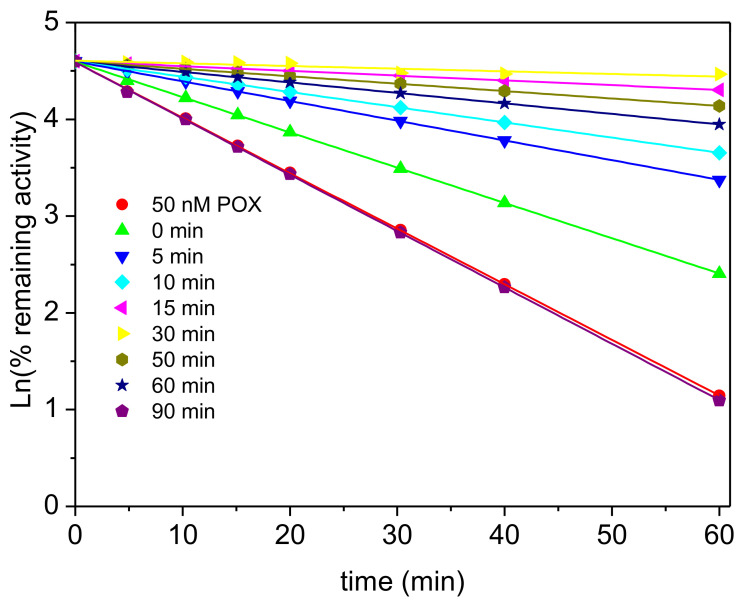
First-order inhibition of AChE by 50 nM paraoxon after pre-incubation of the enzyme in the presence of 10 nM TFK up to 90 min, pH 8.0, 25 °C.

**Figure 14:**
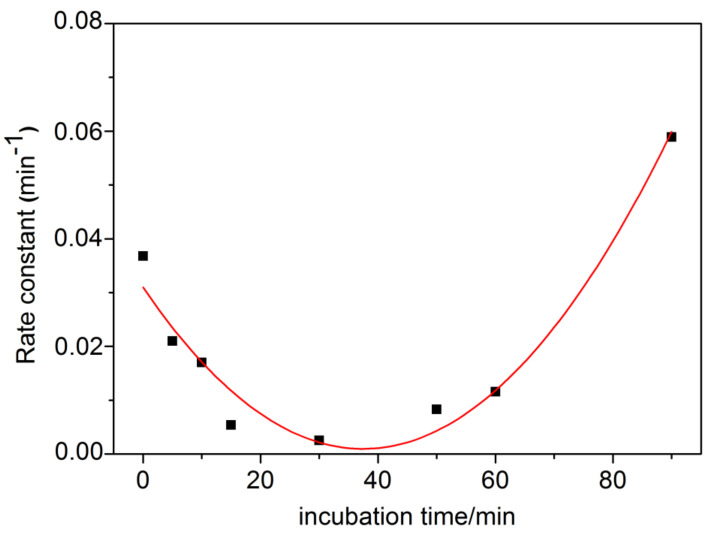
Observed phosphorylation rate constant of hAChE by 50 nM paraoxon as a function of enzyme pre-incubation time (from 0 to 90 min) in the presence of 10 nM TFK.

Then, we investigated the effect of TFK (1–10 nM) on AChE phosphorylation by 50 nM paraoxon. Unlike progressive inhibition of AChE by CSP, first order inhibition of the enzyme by paraoxon is linear as with most OPs. It was found that pre-incubation of AChE with TFK decreased the observed rate of phosphorylation. Thus, the effects of pre-incubation duration and TFK concentration on the rate of inhibition was directly quantified from the slope of *Ln* (residual Activity) vs time and pre-incubation duration the residual activity increased as pre-incubation duration and TFK concentration increased. The protective effect was maximum for 10 nM TFK (Figure 13) and reached a plateau after 40 min pre-incubation (Figure 14).

A recent in vivo work provided evidence that the slow-binding inhibitor C-547 has a long protective action, up to 3 days, on peripheral AChE against inhibition by paraoxon with no side effects [62]. This suggests that C-547 could be used in pre-treatment of OP-poisoning for long-term protection of the peripheral cholinergic system. However, in the case of TFK, the opposite effect on phosphorylation observed between CSP and paraoxon, makes it difficult to predict whether all SBIs can be protectant or anti-protectant of ChEs against all types of OPs. However, it may be hypothesized that the anti-protective effect on AChE reflects the enzyme hysteretic behavior with certain OPs, and results from preferential phosphorylation of the E form that kept memory of SBI binding. A conclusive answer needs further investigation with different SBIs and OPs.

## 4. Conclusions

Kinetic analysis and molecular modeling of hAChE inhibition by TFK showed that this ligand is a SBI of type B. Moreover, after formation of the final complex, a transiently stabilized tetrahedral conjugate is formed, and then slowly dissociates. The existence of covalent and stable acyl tetrahedral intermediates in ChEs [6,63,64] is not completely understood. It is, however, one of the puzzling features of the catalytic power of these enzymes that deserves further studies.

TFK as an SBI of type B capable of binding to human AChE with high affinity, could be of pharmacological interest. It is already the subject of clinical investigations for neuroimaging of neurodegenerative diseases [12]. The related silyl compound, Zifrosilone, a slow tight binding inhibitor of type A with a long residence time *τ* = 70 h and *K_i_* = 0.26 nM for rat brain AChE [7] was promising for symptomatic treatment of AD [7,8,10]. However, human clinical trials were discontinued likely because of its longer residence time on AChE target. Thus, TFK with a much shorter residence time (*τ* ≈ 20 min for reversible binding and overall residence time of about 1 h for full recovery of activity after transient acylation and deacylation of huAChE active serine) appears to be more suitable for further research as an effective and safe pharmacological drug for palliative treatment of AD.

Other possible pharmacological applications may be considered. In particular, research of new molecules for protection of ChEs against phosphylation by OPs is an active field. At the moment, the currently used molecules for pre-treatment of OP poisoning have limited and short protective actions and may induce behavioral and locomotor side effects [65]. Certain SBIs, e.g., huperzine A, galantamine, donepezil have been successfully tested (review in [45]). Novel SBIs may be of interest to expand the duration of AChE protection prior and after exposure to OPs. For example, a recent work from our group provided evidence that the AChE slow-binding inhibitor of type B, C-547, a bulky methyluracil derivative (1,3-bis[5-(diethyl-*o*-nitrobenzylammonium)pentyl]-6-methyluracil dibromide) [45,66] has a long protective action, up to 3 days, on peripheral AChE against its phosphorylation by paraoxon with no side effects [62]. Thus, the low toxicity of TFK in rodents and its protective action on central and peripheral AChEs against toxicity of paraoxon make this compound also of interest for protection of both central and peripheral AChE against OP poisoning (Zueva et al., unpublished).

## Supplementary Materials

Supplementary Materials: The following are available online at https://www.mdpi.com/2218-273X/10/12/1608/s1, Supplementary materials to the Methods section and supplementary figures. Figure S1. Major binding poses found by molecular docking with structures 4EY4-4EY7, 5FPQ as target, clustered together. Representatives of the clusters are colored according to their population from red (most populated) to white (least populated). Figure S2. Individual binding poses of TFK in the PAS from the most populated clusters. Yellow dashes show ordinary hydrogen bonds, cyan dashes show halogen interactions (hydrogen bonds and C-Hal…π interactions), and orange dashes show π-π interactions. Figure S3. Overlay of binding pose of TFK in the PAS shown in details in Figure S2-B (here, TFK molecule is highlightd in magenta) with X-ray structures of mAChE in complex with N-(2-Diethylamino- ethyl)-3-trifluoromethyl-benzenesulfonamide (PDB ID: 4B84, carbon atoms are shown green). Figure S4. Overlay of X-ray structures of hAChE in apo-state (PDB ID: 4EY4, carbon atoms are shown blue) and covalent conjugate with sarin (PDB ID: 5FPQ, carbon atoms are shown green, Ser203-sarin conjugate atoms are shown as spheres). Figure S5. Individual binding poses of TFK in the catalytic active site. Based on docking results with X-ray structures PDB ID 4EY7 (A), 5FPQ (B) and 4EY8 (C) used as a target. Figure S6. Binding of TFK to AChE surface, corresponding to local minima valleys in area 17-18 Å (colored green) and 33-34 Å (colored magenta) from the gorge bottom (see Figure 7, C, blue line for TFK).
