# Supplementary material for: 1-(3-Tert-Butylphenyl)-2,2,2-Trifluoroethanone as a Potent Transition-State Analogue Slow-Binding Inhibitor of Human Acetylcholinesterase: Kinetic, MD and QM/MM Studies
Source: Biomolecules. 2020 Nov 27;10(12):1608. doi: 10.3390/biom10121608 (PMC7760592; doi:10.3390/biom10121608)
Supplement: Supplementary file 1 [file biomolecules-10-01608-s001.pdf]

1-(3-*tert*-butylphenyl)-2,2,2-trifluoroethanone as a potent transition-state analogue slow-binding inhibitor of human acetylcholinesterase: kinetic, MD and QM/MM studies

Irina V. Zueva[1], Sofya V. Lushchekina[2], Ian R. Pottie[3,4], Sultan Darvesh[3,5], Patrick Masson*[6]

[1], Arbuzov Institute of Organic and Physical Chemistry, Federal Research Center "Kazan Scientific Center of the Russian Academy of Sciences", Arbuzov str., 8, Kazan 420088, Russian Federation; [2], Emanuel Institute of Biochemical Physics, Russian Academy of Sciences, Kosygin str. 4, Moscow 119334, Russian Federation;

[3], Mount Saint Vincent University, Department of Chemistry and Physics, Halifax, Nova Scotia, Canada

[4], Saint Mary's University, Department of Chemistry, Halifax, Nova Scotia, Canada

[5], Dalhousie University, Department of Medicine (Neurology and Geriatric Medicine) & Medical Neuroscience, Halifax, Nova Scotia, Canada

[6], Kazan Federal University, Neuropharmacology Laboratory, Kremlevskaya str 18, Kazan 480002, Russian Federation

* to whom correspondence should be addressed (e-mail: pym.masson@free.fr)

Supplementary materials to the Methods section

Targeted molecular dynamics: TMD was used as a tool to build binding pathway. This was the source of starting points for free energy calculations. AChE gorge trafficking is anisotropic, mechanisms of ligand binding and dissociation differ. This was observed experimentally and described at atomic level in computational works [1,2]. Thus, to model binding pathway, it is more correct to pull an inhibitor inside the gorge down to the active site. To ensure that all transformations are performed with the same system, structure of hAChE with inhibitors in unbound state was obtained from the enzyme-inhibitor complex via pulling out the inhibitor and maintaining the initial hAChE structure. More straightforward way would have been to put inhibitor outside the protein and solvate it. However, this would have created difficulties due to different sizes of reference and working systems. The inhibitors were pulled out at distance 25 Å from the initial protein-inhibitor complex (RMSD of the trifluoroacetophenone part of the inhibitors) with force constant 25 kcal/mol·Å^2 in 10 ns MD run, and 5 kcal/mol·Å^2 harmonic restraints applied to C$^\alpha$ and C$^\beta$ atoms, and 0.1 kcal/mol·Å^2 to the other non-hydrogen atoms of hAChE. From this position the inhibitors were pulled into the active site with force constant 5 kcal/mol·Å^2 in 100 ns MD run with the initial structure as target. To avoid protein displacement

caused by pulling force, 5 kcal/mol·Å^2 harmonic restraints were applied to C^α atoms of peripheral amino acids Leu22, Val147, Val226, Leu269, Leu380, Leu414, and Gln474. Pathway outside the protein was also created from this position by pulling further up to 60 Å distance from the initial position. Obtained pathways, pulling the inhibitors inside hAChE active site gorge, and outside the protein, were used to generate initial structures for umbrella-sampling free energy calculations. This procedure is schematically shown in the following figure:

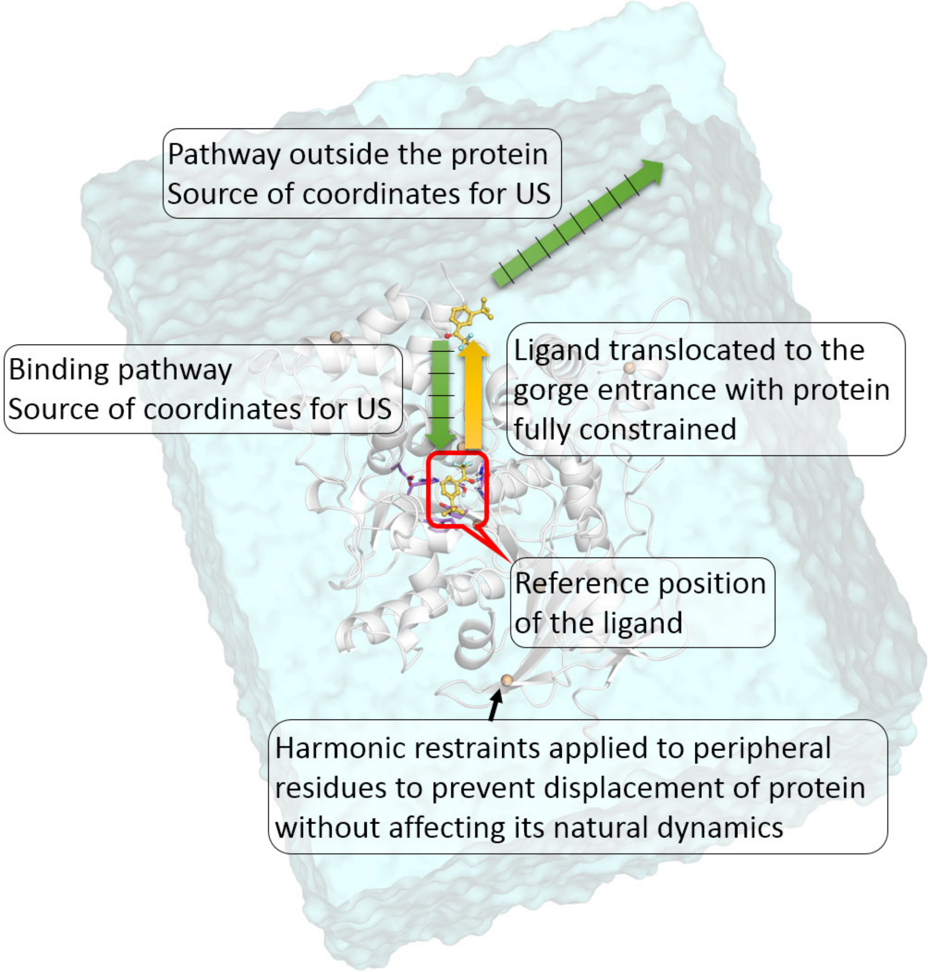

References

1. Xu, Y.; Shen, J.; Luo, X.; Silman, I.; Sussman, J.L.; Chen, K.; Jiang, H. How does huperzine A enter and leave the binding gorge of acetylcholinesterase? Steered molecular dynamics simulations. *J. Am. Chem. Soc.* **2003**, *125*, 11340-11349, doi:10.1021/ja029775t.
2. Kharlamova, A.D.; Lushchekina, S.V.; Petrov, K.A.; Kots, E.D.; Nachon, F.; Villard-Wandhammer, M.; Zueva, I.V.; Krejci, E.; Reznik, V.S.; Zobov, V.V., et al. Slow-binding inhibition of acetylcholinesterase by an alkylammonium derivative of 6-methyluracil: mechanism and possible advantages for myasthenia gravis treatment. *Biochem. J.* **2016**, *473*, 1225-1236, doi:10.1042/BCJ20160084.

Supplementary figures

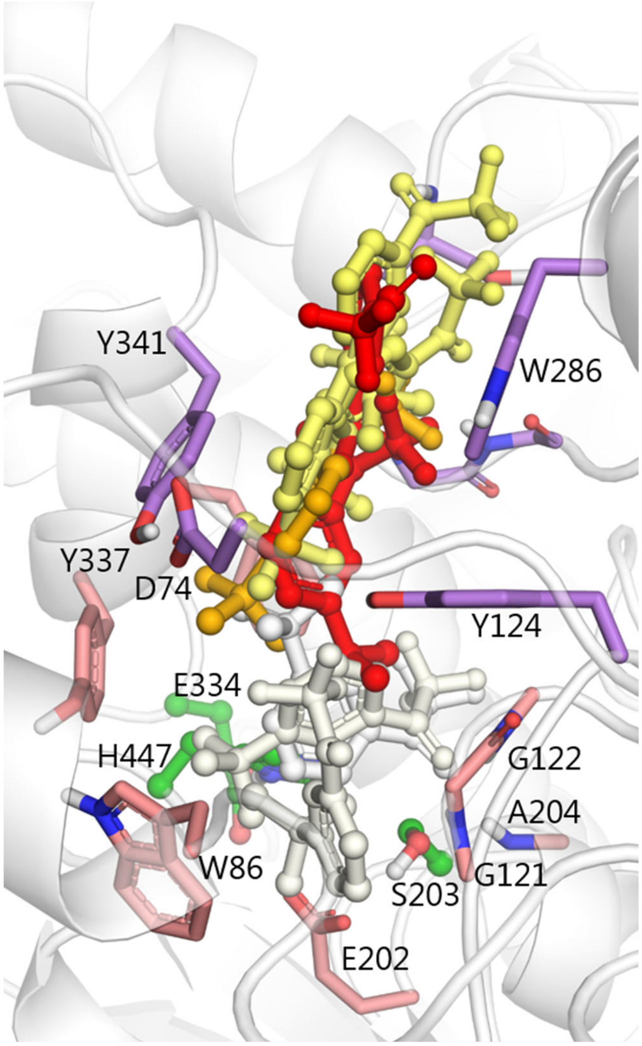

Figure S1. Major binding poses found by molecular docking with structures 4EY4-4EY7, 5FPQ as target, clustered together. Representatives of the clusters are colored according to their population from red (most populated) to white (least populated).

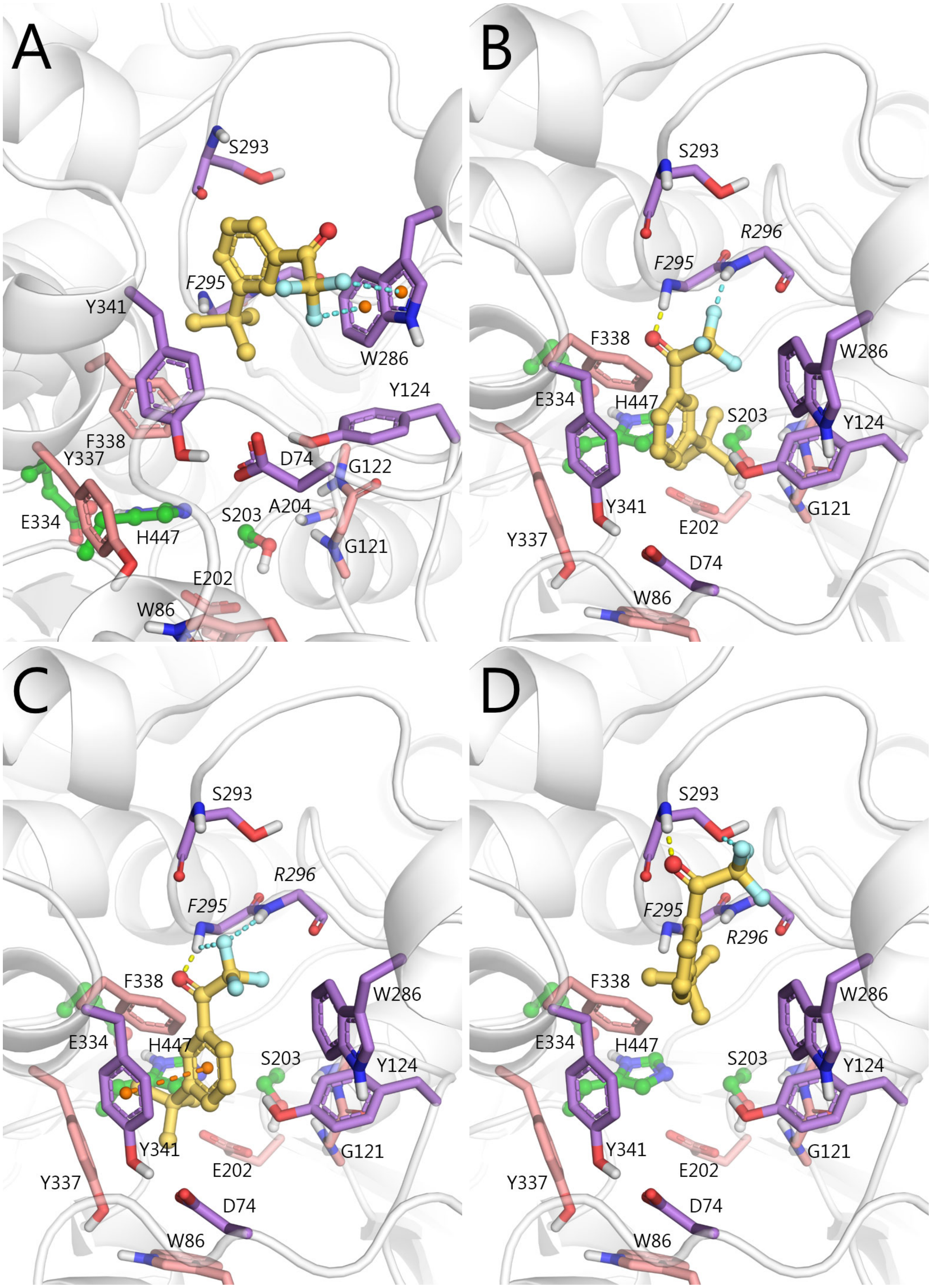

Figure S2. Individual binding poses of TFK in the PAS from the most populated clusters. Yellow dashes show ordinary hydrogen bonds, cyan dashes show halogen interactions (hydrogen bonds and C-Hal…π interactions), and orange dashes show π-π interactions.

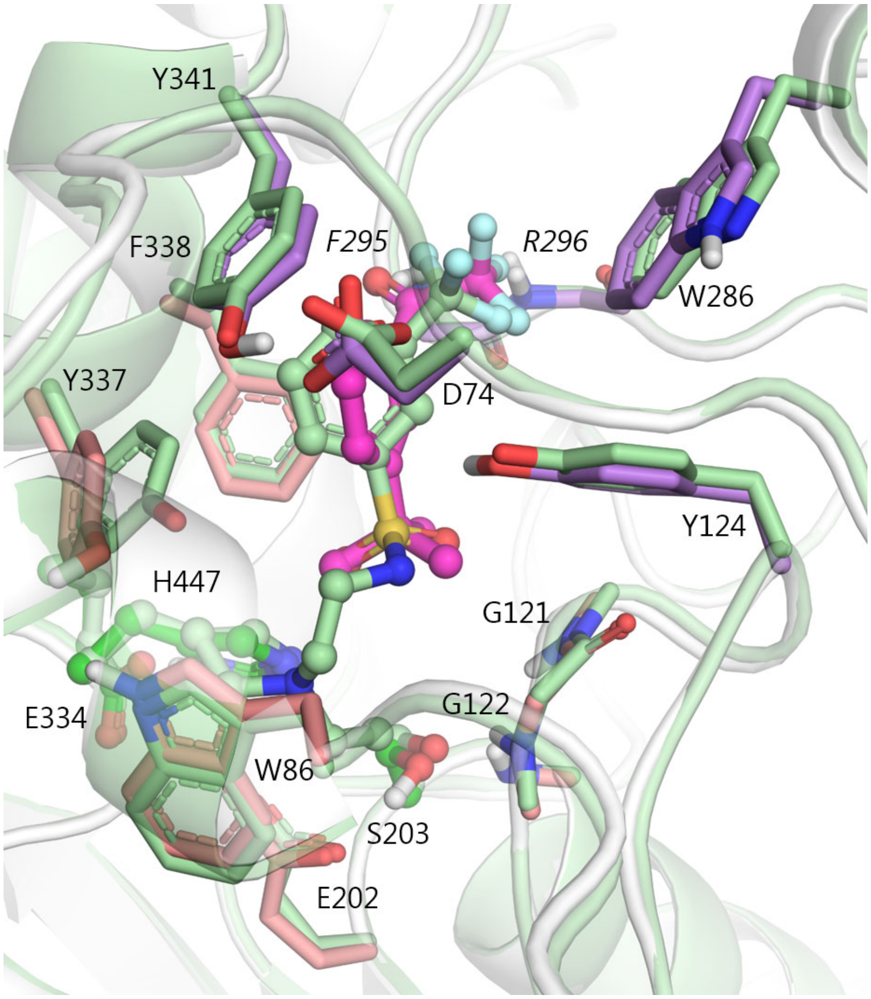

Figure S3. Overlay of binding pose of TFK in the PAS shown in details in Figure S2-B (here, TFK molecule is highlightd in magenta) with X-ray structures of mAChE in complex with N-(2-Diethylamino- ethyl)-3-trifluoromethyl-benzenesulfonamide (PDB ID: 4B84, carbon atoms are shown green).

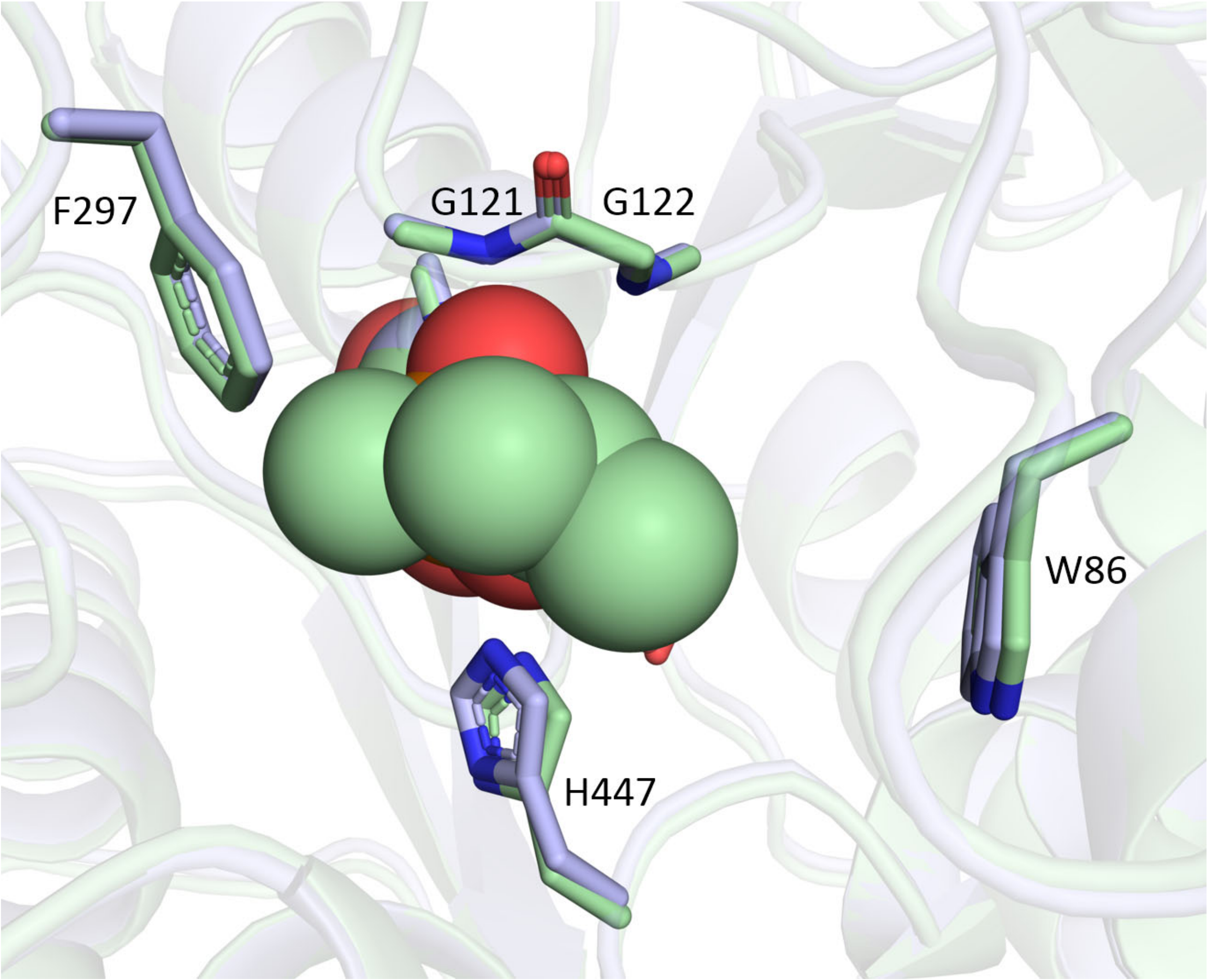

Figure S4. Overlay of X-ray structures of hAChE in *apo*-state (PDB ID: 4EY4, carbon atoms are shown blue) and covalent conjugate with sarin (PDB ID: 5FPQ, carbon atoms are shown green, Ser203-sarin conjugate atoms are shown as spheres).

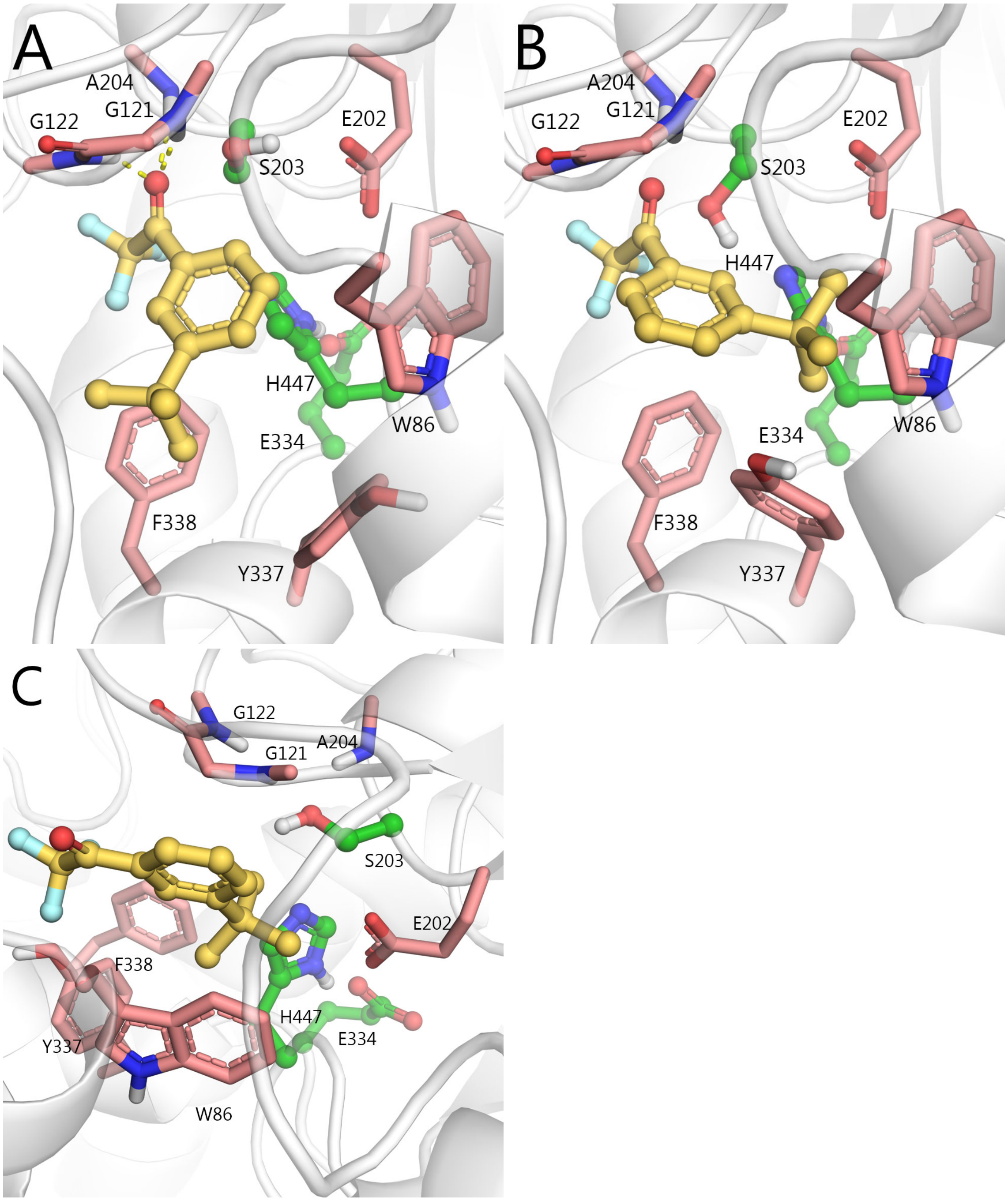

Figure S5. Individual binding poses of TFK in the catalytic active site. Based on docking results with X-ray structures PDB ID 4EY7 (A), 5FPQ (B) and 4EY8 (C) used as a target.

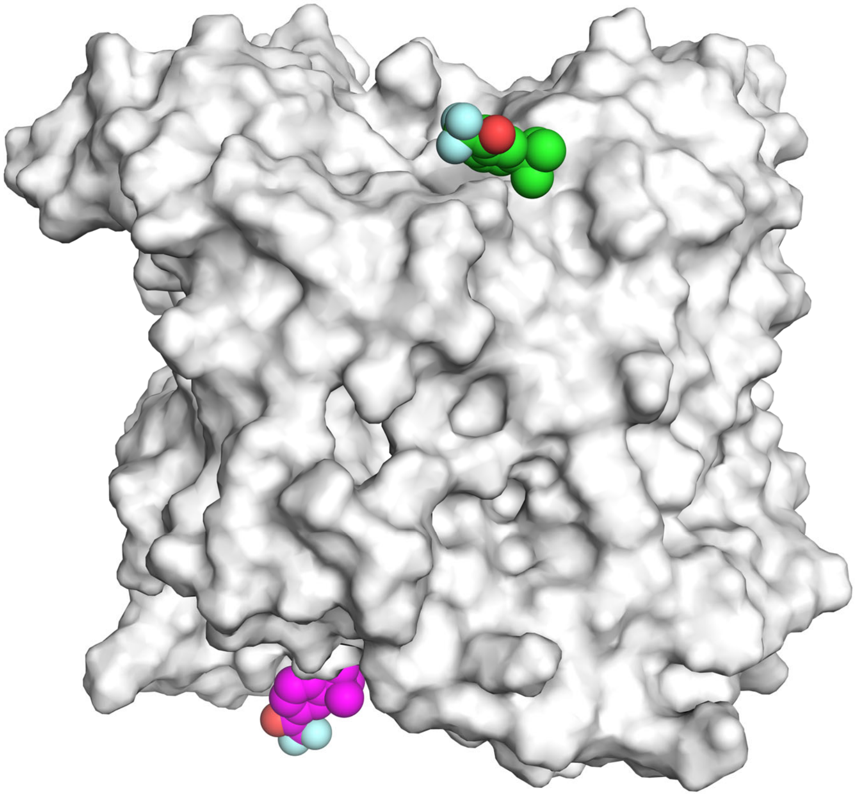

Figure S6. Binding of TFK to AChE surface, corresponding to local minima valleys in area 17-18 Å (colored green) and 33-34 Å (colored magenta) from the gorge bottom (see Figure 7, C, blue line for TFK).
